# Influence of Scleral Buckling Surgery with Encircling Band on Subfoveal Choroidal Thickness in Long-Term Observations

**DOI:** 10.1155/2013/586894

**Published:** 2013-06-11

**Authors:** Dominik Odrobina, Iwona Laudańska-Olszewska, Piotr Gozdek, Mariusz Maroszyński, Michael Amon

**Affiliations:** ^1^Ophthalmology Clinic of St. John Boni Frates Lodziensis, Ulica Kosynierów Gdyńskich 17, 94-049 Lodz, Poland; ^2^Academic Teaching Hospital of St. John, Johannes von Gott Platz 1, 1020 Vienna, Austria

## Abstract

*Purpose*. The aim of this study is the presentation of subfoveal choroidal thickness with enhanced depth imaging spectral-domain optical coherence tomography (EDI-OCT) several months after scleral buckling with encircling band surgery. *Methods*. 48 patients who underwent scleral buckling with encircling band surgery for unilateral rhegmatogenous retinal detachment were included in the retrospective observational study. The mean time from scleral buckling surgery to the final EDI-OCT examination was 22±6.7 months. We compare choroidal thickness between operated and fellow eyes. *Results*. In all patients, the macula was detached before the surgery. The subfoveal choroidal thickness in 48 treated eyes was 260.9±45.8 **µ**m (range 155–383 **µ**m) and in the fellow eyes was 217.5±36.7 **µ**m (range 98–326 **µ**m). The subfoveal choroidal thickness of eyes after scleral buckling surgery in long-term EDI-OCT examination was significantly thicker (*P*<0.001) than in fellow eyes. *Conclusions*. The subfoveal choroid in eyes undergoing encircling band surgery was significantly thicker than in fellow eyes. We suspect that this may be the result of reduced choroidal blood flow. It also seems that the width and size of the material used in scleral buckling surgery may affect a change in the choroid circulation and increase subfoveal choroidal thickness.

## 1. Introduction

 Scleral buckling is a widely used and accepted method for the treatment of rhegmatogenous retinal detachment (RRD). The high percentage of anatomical successes suggests that this method is relatively safe [1, 2].

 Due to rapid developments in modern imaging methods, it is possible to understand changes in the retina better and their influence on functional results after a successful surgery. However, we still know very little about changes in the subfoveal choroid after scleral buckling surgery. Laser Doppler flowmetry has shown a reduced choroidal blood flow in the fovea region after scleral buckling procedures [3]. Changes in choroidal circulation can affect the thickness of the subfoveal choroid [4].

 Enhanced depth imaging- (EDI-) OCT has provided a means to directly image the choroid [5]. The aim of this study is to investigate the changes in subfoveal choroidal thickness following scleral buckling in long-term (more than 6 months) observation with use of an encircling band using EDI-OCT.

## 2. Materials and Methods

This is a retrospective, observational study of 48 patients who underwent scleral buckling surgery with an encircling band for unilateral rhegmatogenous retinal detachment (RRD). Patients were excluded if they had undergone a previous ocular surgery other than uncomplicated cataract surgery or had glaucoma, retinal vascular disorders, macular degeneration, uveitis and other ocular disorders, or high myopia exceeding −6,0 diopters.

 All patients were interviewed, and an ophthalmologic examination was performed before surgery. Examination included BCVA using standard Snellen eye charts, intraocular pressure, anterior segment, and fundus examination with Volk 78 and 90 lenses (Volk Optical Inc., Ohio, USA).

All surgeries were performed by a single surgeon (D.O.) under local anesthesia. Retinal breaks were identified in all patients and were treated with transscleral cryotherapy. A 3.5 mm encircling band (Style 41 DORC; Zuidland, The Netherlands) was used and sutured with 5–0 polyester. Subretinal fluid was drained externally if necessary. No vortex veins and muscles were damaged during operation.

Postoperatively, all eyes were followed up at regular controls. Examination included BCVA using standard Snellen eye charts, intraocular pressure, anterior segment, and fundus examination with Volk 78 and 90 lenses.

**Figure 1:**
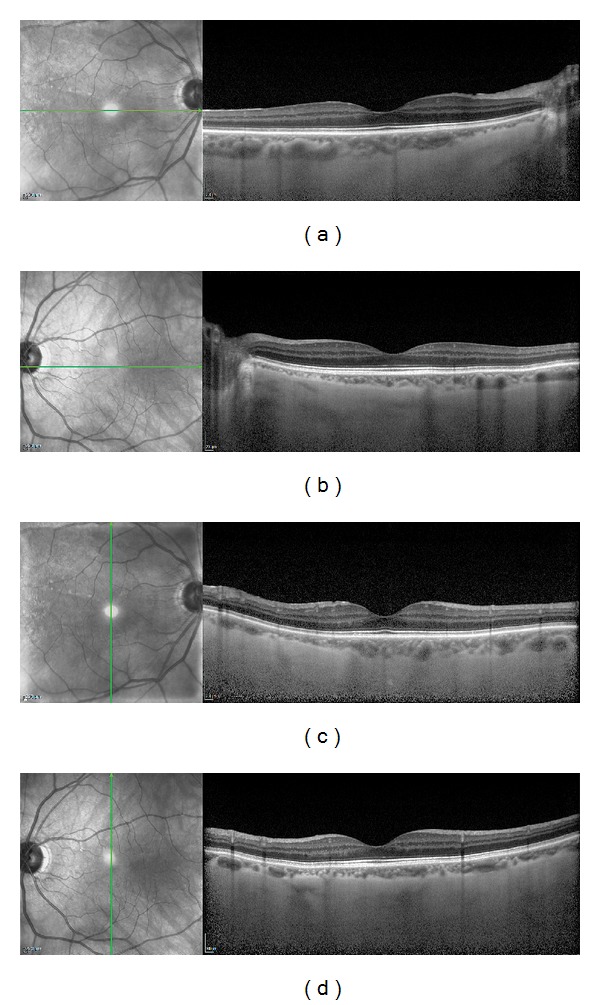
Enhanced depth imaging optical coherence tomography (EDI-OCT) images of a patient 29 months after scleral buckling surgery with encircling band for primary rhegmatogenous retinal detachment. A postoperative EDI-OCT image of a horizontal scan (a) and of a vertical scan (c) of the operated eye. A postoperative EDI-OCT image of a horizontal scan (b) and of a vertical scan (d) of the fellow eye.

Choroidal thickness was measured using EDI-OCT imaging (Spectralis; Heidelberg Engineering, Heidelberg, Germany). It enables us to achieve high definition cross-sectional images of the choroid in vivo. EDI-OCT was performed and analyzed in all patients not earlier than (minimum) 7 months after scleral buckling surgery (range 7–31 months). The subfoveal choroidal thickness was also measured in the fellow eyes (48 eyes) without any previous ocular surgery and any diseases. We compare choroidal thickness between operated and fellow eyes. In each patient we performed two horizontal and vertical line scans through the fovea (Figure 1). In the experiment we derived a medium value from three measurements performed for each patient. Each EDI-OCT examination was done between 11 am and 1 pm. Choroidal thickness was measured in horizontal and vertical sections beneath the fovea. Choroidal thickness was measured manually using manual calipers.

 We characterized the fovea in SD-OCT as the characteristic foveal depression, where there is a lack of the following retinal layers: nerve fibre layer, ganglion cell layer, inner nuclear layer, and inner plexiform layer.

The choroid thickness was measured as the distance between the hyperreflective line corresponding to the base of retinal pigment epithelium (RPE) and the margin or hyperreflective line corresponding to chorioscleral interface.

We compared the manual measurement performed by 2 ophthalmologists, and the results did not differ more than 5 *μ*m and were repeatable. We have taken an average of these two measurements.

 Statistical analyses were carried out using the Mann-Whitney-Wilcoxon rank-sum test for independent data and Wilcoxon signed-rank test for matched pairs. The statistical procedures were performed by the use of Stata Special Edition software, release 12.1 for Windows (StataCorp LP, College Station, TX USA). The significance level was set to be *P* ≤ 0.05.

## 3. Results

The mean age of 48 patients was 59.6 ± 14.6 years (Table 1). Mean preoperative LogMAR visual acuity was 1.76 ± 1.02.

 Retinal detachment was noted in 17 females and in 31 males, in the right eye in 25 cases, and in the left eye in 23 cases. In all patients, the macula was detached before the surgery.

 Anatomic success (complete retinal attachment) was noted in all cases after one surgical intervention. During the last follow-up examination, the retina was still attached in all 48 eyes. The mean LogMAR visual acuity at the final follow-up visit was 0.54 ± 0.31 (*P* < 0.001).

 The mean time from scleral buckling surgery with an encircling band to the final EDI-OCT examination was 22 ± 6.7 months.

 The subfoveal choroidal thickness measured in 48 treated eyes was 260.9 ± 45.8 *μ*m (range 155–383 *μ*m). The subfoveal choroidal thickness in fellow eyes (48 eyes) was 217.5 ± 36.7 *μ*m (range 98–326 *μ*m). The subfoveal choroidal thickness of eyes after scleral buckling surgery in long-term EDI-OCT examination was significantly thicker (*P* < 0.001) than in fellow eyes without any ocular surgeries and any diseases.

## 4. Discussion

In this study we found that the subfoveal choroidal thickness of eyes after scleral buckling surgery using an encircling band, in long-term observation, was significantly thicker (*P* < 0.001) than in fellow eyes.

 To date, few reports have shown changes in choroidal thickness after retinal detachment surgery using the segmental scleral buckling method [6, 7]. Although it has been found that the scleral buckling surgery causes changes in the choroid and retinal microcirculation, the mechanism of these changes is still unclear [3, 6–8].

 Our findings were in contradistinction to the findings of studies of choroidal thickness following segmental scleral buckling [6, 7]. 

 Few authors have analyzed choroidal thickness after scleral buckling surgery, and their observations were carried out only after segmental scleral buckling without use of an encircling band [6, 7]. Their results have shown that subfoveal choroidal thickness increased temporarily after segmental scleral buckling surgery and then returned to postoperative choroidal thickness one to three months after the operation. They found that the greatest increase in thickness of the subfoveal choroid occurs one week after surgery. Kimura and associates did not observe any significant differences in choroidal thickness between treated eyes and fellow eyes before or three months after operation. Miura and associates also showed similar observations before surgery, but choroidal thickness returned to normal one month after surgery.

 The thickness of the subfoveal choroid or subfoveal choroidal blood flow is unchanged or can return to its initial state, but the mechanism of these changes is still unclear [7, 8]. Takahashi and Kishi showed in the indocyanine angiography venovenous anastomoses formation the remodeling of the choroidal venous drainage more than three months after surgery for retinal detachment [9]. In our study, subfoveal thickness was significantly thicker (*P* < 0.001) than in fellow eyes. Our EDI-OCT observation was performed also more than 3 months (22 ± 6.7 months) after surgery. These results may suggest that the mechanism of changes in the choroid circulation is different after scleral buckling surgery with or without an encircling band.

 The encircling band causes changes in the circulations of both the retina and the choroid. When increasing the thickness of the subfoveal choroid in the long-term observations after surgery, we found indications of persistent changes in choroid circulation. Although previous studies state that scleral buckling surgery reduces choroidal blood flow, the results are not consistent. Our results were similar to those of Ito and associates, who found changes in choroid circulation without returning to the baseline after scleral buckling surgery [10]. In their studies, Kimura and associates also observed the fact that the encircling band causes a reduction of choroidal blood flow [11]. Choroidal circulation has improved among patient who had caused a relaxation of the encircling band.

 Scleral buckling reduces blood flow and increases hemostasis in choroidal circulation. This may cause an elevation in choroidal blood pressure and may increase the subfoveal choroidal thickness. Because of the fact that the encircling band causes compression of a 360 degree circular area, choroid blood flow has been changed to a much larger area of the eye than in the case of segmental scleral buckling. Our results are different from those of other authors who reported that the subfoveal choroid is thicker in the initial period just after the surgery. However, Kimura et al.'s study [6] only analyzed cases in which the size of the scleral buckling was 107 ± 38.8 degrees, and only in one patient in Miura et al.'s study [7] the extent of scleral buckling was 360 degrees. The size of the scleral buckling may therefore have an impact on choroidal circulation over a longer period of time and may reduce the possibility of choroidal thickness returning to the baseline.

 It seems that the width of the material may also affect the change in the choroid circulation according the rule of physics, *P* is *F*/*A* (*P* is pressure, *A* is area of the surface, *F* is force acting normally to the surface). In our study we used an encircling band with a width of 3.5 mm. Kimura and associates and Miura and associates used a silicone sponge with a width of 5 mm and a silicone tire with a width of 7 mm [6, 7]. Using an implant with smaller width and using the same force, the formation of stitches obtained more pressure on the eye, as compared with an implant of greater width (*P* = *F*/*A*). The encircling band can cause vascular stasis in the choroidal circulation due to the venous obstruction and their reduced drainage.

 Another factor that can change the subfoveal choroidal thickness is periocular inflammation. Veckeneer et al. showed that the aqueous flare was higher after cryotherapy than after laser photocoagulation [12]. Cryotherapy used during surgery can cause scleral and choroidal inflammation, which may increase the thickness of the choroid [6]. However, this applies only in the early postoperative period [6, 7].

 The limitation of this study is lack of data before surgery. This data could give us information on whether retinal detachment itself does not cause changes in choroidal thickness in the foveal area. However, it is difficult to perform good quality EDI-OCT in eyes where the macula was detached before the surgery. Another limitation of this study is the wide variation of the observation period.

## 5. Conclusions

In conclusion, the subfoveal choroid in eyes undergoing encircling band surgery was significantly thicker than in fellow eyes without any ocular surgeries or diseases, in long-term observation. This may be caused by a reduced choroidal blood flow and may not return to the initial state. It also seems that the width and size of the material used in scleral buckling surgery may affect a change in the choroid circulation and increase subfoveal choroidal thickness. Further studies are needed to investigate these factors. 

## Figures and Tables

**Table 1:** Descriptive statistics for demographic data, along with subfoveal choroidal thickness and LogMAR measurements in patients undergoing retinal detachment surgery based on scleral buckling with encircling band (*n=48*).

	M^†^	Me^‡^	SD**	SE^††^	95% CI^‡‡^	CV***	Min.–max.	*P*-value
Patients' age (years)	59.69	62.00	14.67	2.12	55.43–63.95	24.57%	24–89	
Observation duration time (months)	22.06	24	6.75	0.97	20.10–24.02	30.59%	7–31

LogMAR* preoperative measurement in the operated eye	1.7697	1.6109	1.0270	0.1482	1.4715–2.0679	58.03%	0.3010–4	<0.001
LogMAR measurement in the operated eye during the final examination	0.5439	0.3979	0.3182	0.0459	0.4515–0.6363	58.51%	0.1549–1.3010	

Subfoveal choroidal thickness in the operated eye during the final examination	260.90	261	45.88	6.62	247.57–274.22	17.58%	155–383	<0.001
Subfoveal choroidal thickness in the fellow eye during the final examination	217.56	224	36.71	5.30	206.90–228.22	16.87%	98–326	

*LogMAR: Logarithm of the Minimum Angle of Resolution.

^†^M: mean value.

^‡^Me: median value.

**SD: standard deviation.

^††^SE: standard error.

^‡‡^CI: confidence interval.

***CV: coefficient of variation.
